# *Pseudomonas aeruginosa* Exolysin promotes bacterial growth in lungs, alveolar damage and bacterial dissemination

**DOI:** 10.1038/s41598-017-02349-0

**Published:** 2017-05-18

**Authors:** Stéphanie Bouillot, Patrick Munro, Benoit Gallet, Emeline Reboud, François Cretin, Guillaume Golovkine, Guy Schoehn, Ina Attrée, Emmanuel Lemichez, Philippe Huber

**Affiliations:** 1 Université Grenoble Alpes, F-38000 Grenoble, France; 2 CNRS, ERL5261, F-38000 Grenoble, France; 3 CEA, BIG-BCI, F-38000 Grenoble, France; 4 INSERM, U1036, F-38000 Grenoble, France; 5 INSERM, U1065, F-06204 Grenoble, France; 6 Université de Nice-Sophia-Antipolis, F-06204 Grenoble, France; 7 CEA, IBS, F-38000 Grenoble, France; 8 CNRS, UMR5075, F-38000 Grenoble, France

## Abstract

Exolysin (ExlA) is a recently-identified pore-forming toxin secreted by a subset of *Pseudomonas aeruginosa* strains identified worldwide and devoid of Type III secretion system (T3SS), a major virulence factor. Here, we characterized at the ultrastructural level the lesions caused by an ExlA-secreting strain, CLJ1, in mouse infected lungs. CLJ1 induced necrotic lesions in pneumocytes and endothelial cells, resulting in alveolo-vascular barrier breakdown. Ectopic expression of ExlA in an *exlA*-negative strain induced similar tissue injuries. In addition, ExlA conferred on bacteria the capacity to proliferate in lungs and to disseminate in secondary organs, similar to bacteria possessing a functional T3SS. CLJ1 did not promote a strong neutrophil infiltration in the alveoli, owing to the weak pro-inflammatory cytokine reaction engendered by the strain. However, CLJ1 was rapidly eliminated from the blood in a bacteremia model, suggesting that it can be promptly phagocytosed by immune cells. Together, our study ascribes to ExlA-secreting bacteria the capacity to proliferate in the lung and to damage pulmonary tissues, thereby promoting metastatic infections, in absence of substantial immune response exacerbation.

## Introduction

*Pseudomonas aeruginosa* is a Gram-negative bacterium and a major opportunistic pathogen causing nosocomial infections^1, 2^. This bacterium can engender acute infections in patients with implanted medical devices, such as ventilators, blood or urinary catheters, or with wounds, burns and keratitis. *P. aeruginosa* is also responsible of chronic infections in patients with cystic fibrosis or chronic obstructive pulmonary disease (COPD), leading to high morbidity and fatality rate. Its virulence relies on various regulated factors acting in concert to allow the bacteria to penetrate or colonize tissues. The bacterium’s most potent toxins are those secreted directly into the host cytoplasm by a Type III Secretion System (T3SS). These toxins (ExoU, ExoS, ExoT, ExoY) have a striking effect on host cells by inducing plasma membrane rupture or actin cytoskeleton disruption, depending on the injected toxins^3–5^. In general, ExoU and ExoS, are mutually exclusive, with approximately 30% of ExoU^+^ strains among clinical isolates. The most potent toxin ExoU possesses a phospholipase A2 activity targeting the plasma membrane^4^. *In vivo*, the pivotal role of the T3SS and its effectors has been established in mouse models of infection and by the analysis of numerous clinical strains in correlation with the severity of patients’ disease^6–10^.

Recently, we characterized a clinical strain, called CLJ1, isolated from a patient suffering from COPD associated with haemorrhagic pneumonia, that was cytolytic in cellular models and hypervirulent in the mouse^11^. CLJ1 is devoid of T3SS, because the genes encoding the proteins of the T3SS and its effectors are lacking in this strain. CLJ1 virulence relies on a pore-forming toxin, called Exolysin or ExlA, whose sequence is quite homologous (35% identity) to that of ShlA from *Serratia marcescens*. For secretion in the extracellular milieu, ExlA requires *exlB*, a gene upstream of *exlA*, encoding a potential porin located in bacterium’s outer membrane^11^. ExlA/ExlB, encoded by the *exlBA* locus, constitutes a two-partner secretion system. Other strains lacking the T3SS genes and containing *exlBA* (hereafter *exlA*+ strains) were identified in various *P. aeruginosa* collections. They have been isolated from patients with several types of infection diseases, and in five continents, suggesting that *exlA*+ strains have propagated worldwide^12, 13^.

In a mouse model of pneumonia, strains of *P. aeruginosa* expressing ExlA displayed a virulence that correlated with levels of ExlA secretion^13^. *ExlA*+ strains, specifically those secreting high levels of ExlA, displayed toxicity on various cell types, including epithelial, endothelial, fibroblastic and myeloid cells, but had little effect on erythrocytes^13^. ExlA induces host plasma membrane permeability, as evaluated by lactate dehydrogenase (LDH) release and ethidium bromide incorporation, hence demonstrating its capacity to induce necrotic cell death^11^. However, the lesions induced by ExlA in the infected lungs, the capacity of the toxin to allow bacterial survival and growth in lung or blood, or to promote dissemination in various organs remained to be characterized.

Here, we examined the survival and growth of CLJ1, the most virulent *exlA*+ strain, in mice subjected to pneumonia or bacteremia. This strain maintained a high capacity to secrete ExlA, probably due to few passages on laboratory media. Engineered laboratory strains *T3SS*− *exlA*+ or *T3SS*− *exlA*− were also used to evaluate the direct role of ExlA in these models. ExlA or the T3SS allowed survival and growth of bacteria in lung, while bacteria devoid of either virulence factors were unable to proliferate in this compartment. Interestingly, bacterial survival in the blood, mainly depended upon the genetic background of the strain rather than T3SS or ExlA expression. Bacterial dissemination from the lung was monitored by numeration of viable bacteria in various organs at different times post-infection. We show that the main target organs are the liver and spleen, and that ExlA or the T3SS are required for invasion. Interestingly, the brain was colonized independently of ExlA or T3SS. Electron micrographs of lungs infected with ExlA-secreting strains induced more prominent tissue damage and necrosis, compared to strains injecting exotoxins S, T and Y, or without T3SS. Altogether, our results show that ExlA is a necrotizing toxin endowed with the capacity to promote bacterial invasion from the lung and colonisation of secondary organs.

## Results

### Bacterial load in infected mouse lungs

**Figure 1:**
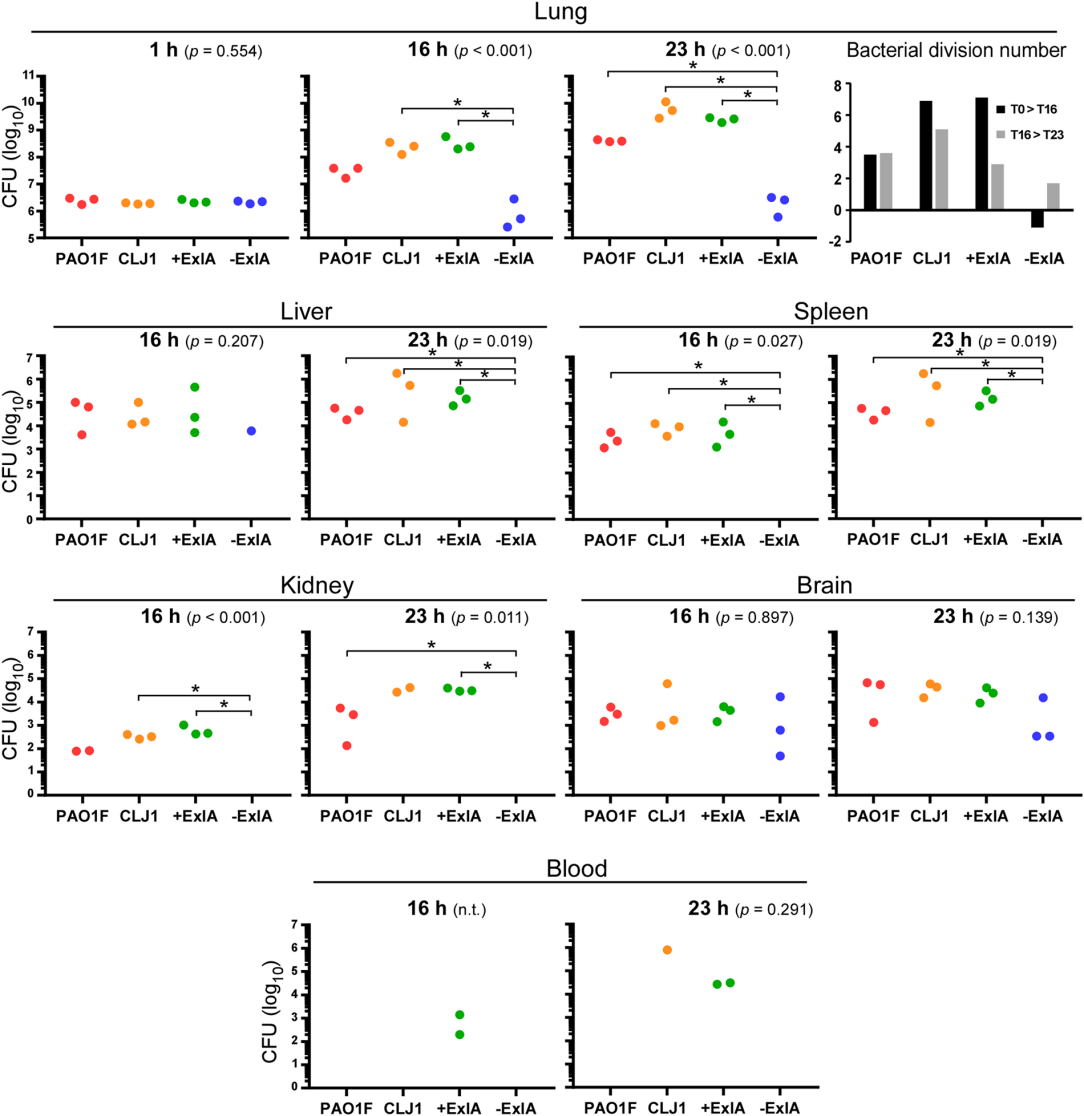
Pulmonary infection: bacterial load in the lung and dissemination in various mouse organs. Mouse lungs were infected with 2.5 × 10^6^ bacterial suspensions of PAO1F, CLJ1, PAO1FΔT3SS::*exlBA* (+ExlA), PAO1FΔT3SS::empty vector (−ExlA). Organs (lung, spleen, liver, kidney, brain and blood) were isolated at 1, 16 and 23 h.p.i., as indicated, were homogenized and serial dilutions of the homogenates were plated onto agar plates to determine the CFU per organ. Of note, no bacteria were found outside of the lung at 1 h.p.i. Data are represented by solid circles on logarithmic scales. Three mice were used per strain and per time point. Statistical differences were calculated using Kruskal-Wallis’s test and probabilities are indicated between parenthesis in each condition; n.t., not testable; pairwise comparisons were established with Holm-Sidak’s post-hoc test: *p < 0.05. In the lungs, the bacterial division rates between 1 and 16 h.p.i. (T1 > T16), and between 16 and 23 h.p.i. (T16 > T23), were calculated for the different strains and plotted (upper right).

The T3SS was previously shown to be required for *P. aeruginosa* survival in the lungs and efficient infection^6, 9, 10^. To assess the survival capacity of ExlA-positive strains in the lungs, we examined the evolution of this bacterial population after intranasal inhalation of 2.5 × 10^6^ bacteria from CLJ1 *exlA*+ strain (Fig. 1). As CLJ1 cannot be manipulated genetically^11^, to address the direct function of ExlA in mice, we generated the PAO1FΔ*T3SS*::*exlBA* (hereafter +ExlA), a strain devoid of T3SS but secreting ExlA in similar amount as CLJ1^11^, and PAO1FΔ*T3SS*::empty vector (hereafter −ExlA), an isogenic control strain lacking the T3SS and ExlA. We also included the parental strain PAO1F, a *P. aeruginosa* strain secreting three T3SS effectors: ExoS, ExoT and ExoY. Mice were euthanized at 1, 16 and 23 hours post-infection (h.p.i.). At 1 h.p.i., the bacterial loads in lung tissues were similar to the initial inoculum (2.5 × 10^6^), indicating that the bacteria reached the lungs, but did not start to proliferate. At 16 and 23 h.p.i., the populations of CLJ1, PAO1F and +ExlA were dramatically expanded, particularly for CLJ1 and +ExlA strains (see the calculated bacterial division numbers in Fig. 1). Conversely, the bacterial population of −ExlA did not grow or slightly decreased during this time, while it did not display slower growth rates in LB medium compared to other strains (Supplementary Fig. 1). Thus, expression of ExlA in *P. aeruginosa* lacking a T3SS confers to the bacterium a major virulence advantage, i.e. bacterial growth and survival rates in lung tissues.

### Bacterial dissemination from the lung

Previous studies established the capacity of CLJ1 to disseminate in the spleen of mice infected via a pulmonary route^11^. We went on to assess bacterial dissemination to other organs. Bacterial dissemination was evaluated by CFU counting in spleen, liver, kidney, brain and in blood (Fig. 1). No bacteria could be detected within these organs at 1 h.p.i. (not shown). At 16 and 23 h.p.i., the liver and spleen contained the highest disseminated populations of CLJ1, PAO1F and +ExlA, followed by brain and kidney. No −ExlA bacteria were found in spleen, liver and kidney, except for one mouse in liver. Surprisingly, −ExlA bacteria were detected in the brain of all six infected mice. Thus, −ExlA bacteria only homed in the brain, as opposed to the other strains, which colonized a broader spectrum of organs. Bacteria from all strains were rarely detected in the blood, suggesting that they did not remain in this bactericidal environment, but rapidly accumulated in target organs (see below).

No morphological alterations of secondary infected organs were observed by hematoxylin/eosin staining of tissue sections at 18 h.p.i. (data not shown), except for the spleens of mice infected by PAO1F, CLJ1 and +ExlA, displaying red pulp hyperaemia, white pulp hyperplasia and B lymphocyte apoptosis, clearly indicating that spleens were immunoreactive at that time (see Supplementary Fig. 2). The localization of disseminated bacteria was determined by immunohistological analysis of tissue sections (see Supplementary Fig. 3). Isolated or aggregated bacteria were found in hepatic lobules, white pulp and marginal zones of spleen, intertubular space of kidney and brain parenchyma, indicating that bacteria penetrated deeply (i.e. in the retro-endothelial compartment) in these organs. No difference in localisation were found between the strains.

### ExlA induces major injury in the lung

CLJ1 induces plasma membrane permeability in host cells, a process that can be monitored by lactate dehydrogenase (LDH) release in the extracellular medium^13^. Here, we first followed the fate of CLJ1-infected A549 cells by real-time microscopy. CLJ1 induced cell rounding, starting from 180 min p.i., followed by plasma membrane rupture from 210 min p.i. onward (as illustrated in Supplementary Fig. 4). Plasma membrane rupture triggered the immediate release of the cytosolic components in the extracellular medium, as seen by the release of GFP.

**Figure 2:**
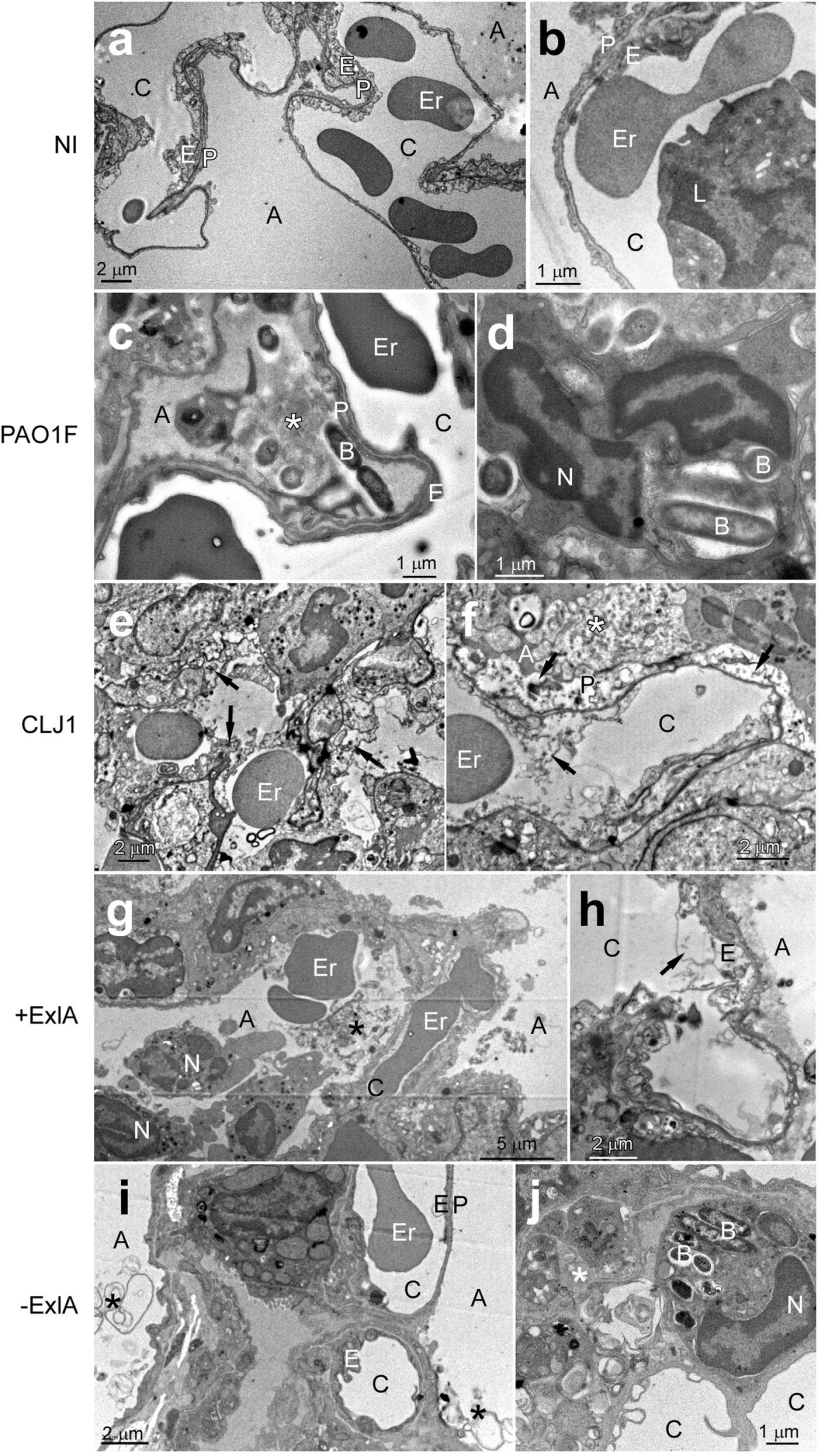
Electron micrographs of infected lungs. (**a**–**j**) Mice (n = 2 per strain) were infected with 5 × 10^6^ bacteria from PAO1F, CLJ1, PAO1FΔT3SS::*exlBA* (+ExlA) or PAO1FΔT3SS::empty vector (−ExlA) strains, or were uninfected (NI), as indicated (2 images per condition). Mice were euthanized at 18 h.p.i. and lungs were isolated and prepared for electron microscopy. Abbreviations: A, alveolus; B, bacterium; C, capillary; E, endothelial cell; Er, erythrocyte; L, leukocyte; N, neutrophil; P, pneumocyte, *intra-alveolus material: mucus, cellular debris, surfactant. Arrows indicate endothelial or epithelial necrosis.

To analyse the effect of CLJ1 on pulmonary tissues and the contribution of ExlA to tissue damage, we observed sections of infected mouse lungs by transmission electron microscopy (TEM) (Fig. 2). Control lung images are shown in Fig. 2a,b. A very dense pus with numerous bacteria and neutrophils were present in the alveoli of PAO1F-infected lungs, but cells in the alveolar wall were not necrotic (Fig. 2c,d). In CLJ1-infected lungs, there were large areas with destruction of the tissue architecture and necrotic alveolar walls (arrows in Fig. 2e,f). The alveoli were filled with pus (indicated by *) containing cell debris, pneumocytic granules, matrix fragments, surfactant, mucus as well as neutrophils. Erythrocytes were observed in the alveoli, in addition to the vascular compartment, indicating that the alveolo-vascular barrier was disrupted. No signs of necrosis were noted in the bronchi (not shown). Similar evidence of necrosis, intra-alveolar erythrocytes, neutrophils and pus were found in +ExlA-infected lungs (Fig. 2g,h). Pus and neutrophils were rarely observed in alveoli of −ExlA-infected lungs, and the architecture of the alveolar walls was preserved (Fig. 2i,j). Interestingly, phagocytosed bacteria were found in intra-alveolar neutrophils of PAO1F- or −ExlA-infected mice, while no images of phagocytosis were observed with the two other strains. Intriguingly, no bacteria could be visualized in CLJ1-infected lungs, while they harboured the highest amounts of CFU at 16 h.p.i. (Fig. 1). CLJ1 bacteria are non-adhesive to any support, as opposed to PAO1F (personal observation). It is thus likely that CLJ1 bacteria were washed out during the numerous washing steps of tissue preparation for electron microscopy, but are still preserved in preparations for conventional microscopy (see Supplementary Fig. 3).

Taken together, ExlA-positive strains induced a striking deterioration of lung alveoli, with dramatic alterations of the pneumocytic and endothelial layers, responsible of erythrocyte spread in the lung and bacterial dissemination in the mouse body. The necrotic figures are reminiscent of what was observed on CLJ1-infected A549 cells (Supplementary Fig. 4).

**Figure 3:**
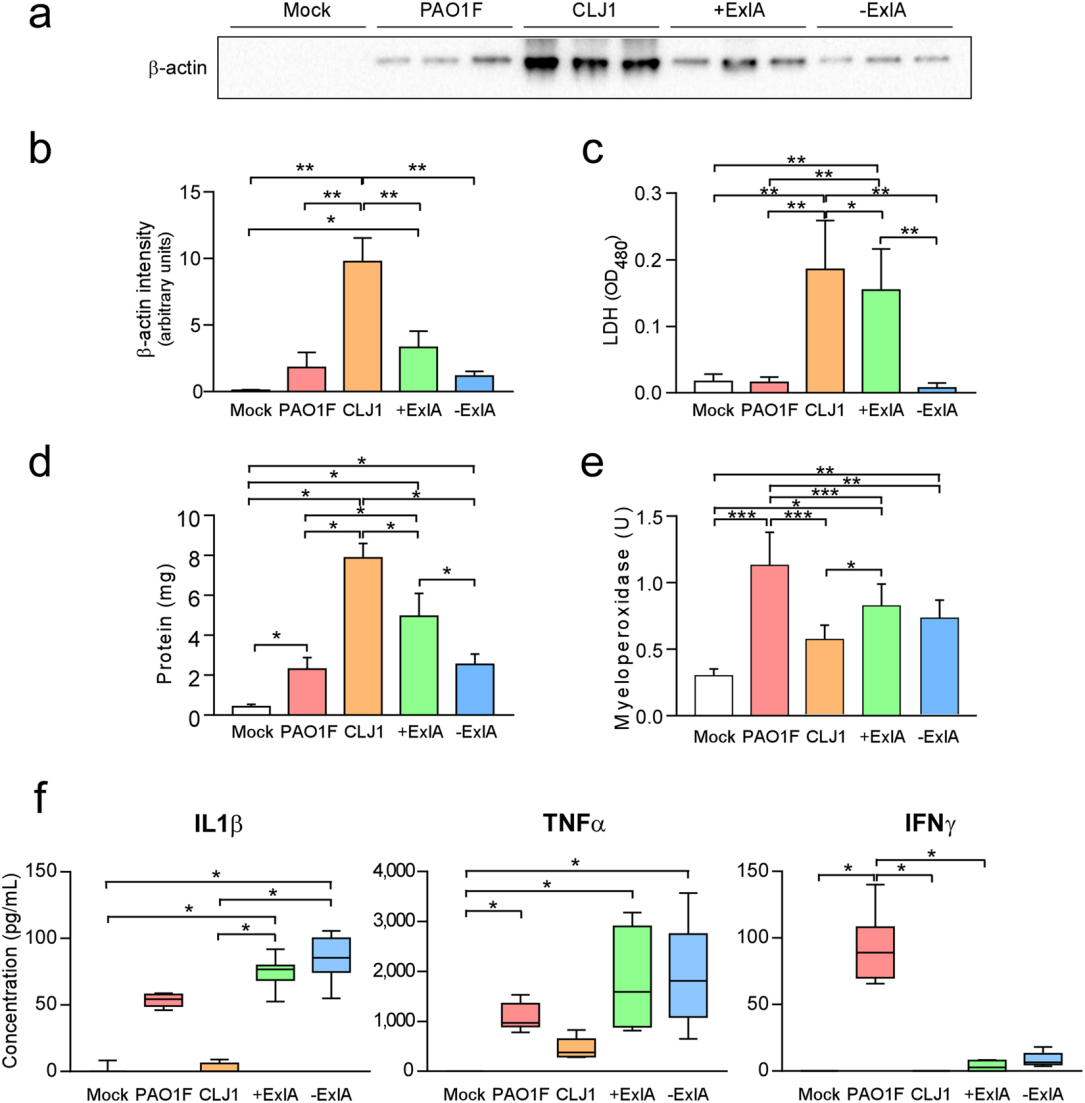
Intracellular proteins and pro-inflammatory cytokines in broncho-alveolar lavages. BALs were sampled at 18 h.p.i. after pulmonary infection with PAO1F, CLJ1, +ExlA or −ExlA (5 × 10^6^), or mock infection with PBS, and were centrifuged to separate the cellular pellet from the soluble proteins. (**a**) Western blot analysis of β-actin in 3 BAL supernatants per condition (representative of 6–8 BALs per condition) and (**b**) histogram of band intensities. Statistics: ANOVA, p < 0.001; Holm-Sidak’s post-hoc test: *p = 0.031, **p < 0.001. (**c**) LDH measurements in 6 BAL supernatants per condition. Statistics: ANOVA, p < 0.001; Holm-Sidak’s post-hoc test: *p = 0.028, **p < 0.001. (**d**) Total proteins in BAL supernatants (n = 6). Statistics: ANOVA, p < 0.001; Holm-Sidak’s post-hoc test: *p < 0.001. (**e**) Myeloperoxidase activity in BAL pellets (n = 6). Statistics: ANOVA, p < 0.001; Holm-Sidak’s post-hoc test: ***p < 0.001, **p = 0.003, *p < 0.05. (**f**) IL-1β, TNF-α, and IFN-γ concentrations were measured in BAL supernatants (n = 6–8). Statistics: Kruskal-Wallis’s test, p < 0.001; Dunn’s post-hoc test: *p < 0.05. IL-10, IL-17 and IL-12p70 dosages yielded negligible values for all strains (not shown).

To further assess necrotic cell death in the lung, we examined the presence of two intracellular proteins, β-actin and LDH, in broncho-alveolar lavages (BAL) fluids at 18 h.p.i. of infected or non-infected mice. BALs of CLJ1-infected mice contained significantly higher amounts of β-actin than any other BALs (Fig. 3a,b). BALs from +ExlA-infected mice also contained significantly higher amounts of β-actin than BALs from mock-infected mice, while BALs from PAO1F or −ExlA-infected mice contained minimal β-actin amounts, not significantly different from mock-infected mice. Similarly, LDH activity was significantly higher in BALs from CLJ1-infected mice than from any other BALs; BALs from +ExlA-infected mice also exhibited higher LDH activity than other BALs except for CLJ1 (Fig. 3c). These data confirm the necrotizing activity of ExlA, as previously observed by TEM in infected lungs (Fig. 2).

The permeability of the alveolo-capillary barrier was assessed by the measurement of BAL protein content (Fig. 3d). CLJ1 and +ExlA induced a major increase of protein content in lung airways, further confirming that the barrier is damaged. This increase in barrier permeability is at least in part due to the presence of ExlA, as −ExlA induced significantly less protein diffusion than +ExlA. As previously reported^14^, PAO1F also significantly increased BAL protein content compared to mock-infected lungs.

### Inflammatory cytokines in broncho-alveolar lavages from lung-infected mice

Rapid neutrophil infiltration in the lung is a hallmark of *P. aeruginosa* pulmonary infections and promotes an efficient response to the pathogen^15^. In the same experiment as above, we measured the myeloperoxidase (MPO; a specific enzyme of neutrophils) activity present in the cellular component of BALs to evaluate neutrophil recruitment in the airways. Strains with PAO1F background all induced a significant increase of MPO activity compared to mock, while the increase was non-significant for CLJ1 (Fig. 3e). These results confirm that neutrophils are moderately recruited upon CLJ1 infection^11^. We thus wondered whether the inflammatory cytokine response was different when mice were infected by either strain. IL (interleukin)-1β, TNF (tumour necrosis factor)-α and IFN (interferon)-γ pro-inflammatory cytokines were not significantly different in BALs from CLJ1- or mock-infected mice (Fig. 3f). On the contrary, PAO1F, +ExlA and −ExlA strains triggered higher IL-1β and TNF-α responses than the negative control (Fig. 3f). We noticed that IFN-γ was only found in BALs from PAO1F-infected lungs (Fig. 3f), not when lungs were infected with +ExlA or −ExlA, suggesting that the T3SS induced IFN-γ release. Neither the pro-inflammatory cytokine IL-12p70, IL-17 nor the anti-inflammatory cytokine IL-10 were detected in BALs from lungs infected by either strains (data not shown). These results indicate that CLJ1 is rather silent in terms of innate immunity responses and that ExlA expression does not profoundly promote inflammatory cytokine responses.

To further examine the effect of CLJ1 on IL-1β expression and on IL-1β maturation by the inflammasome, we infected A549 alveolar cells and analysed the pro-IL-1β content of cell lysates and the presence of IL-1β in cell supernatants (Supplementary Fig. 5). No pro-IL-1β or IL-1β were detected in A549 cells or cell supernatants, respectively, indicating that CLJ1 did not induce IL-1β expression. As controls, strains with PAO1F background all induced pro-IL-1β expression and IL-1β secretion. Thus, the weak IL-1β response in CLJ1-infected mice is likely not due to a lack of inflammasome activation, but to the absence of bacterial determinants inducing pro-IL-1β synthesis.

### Bacterial survival in the blood

**Figure 4:**
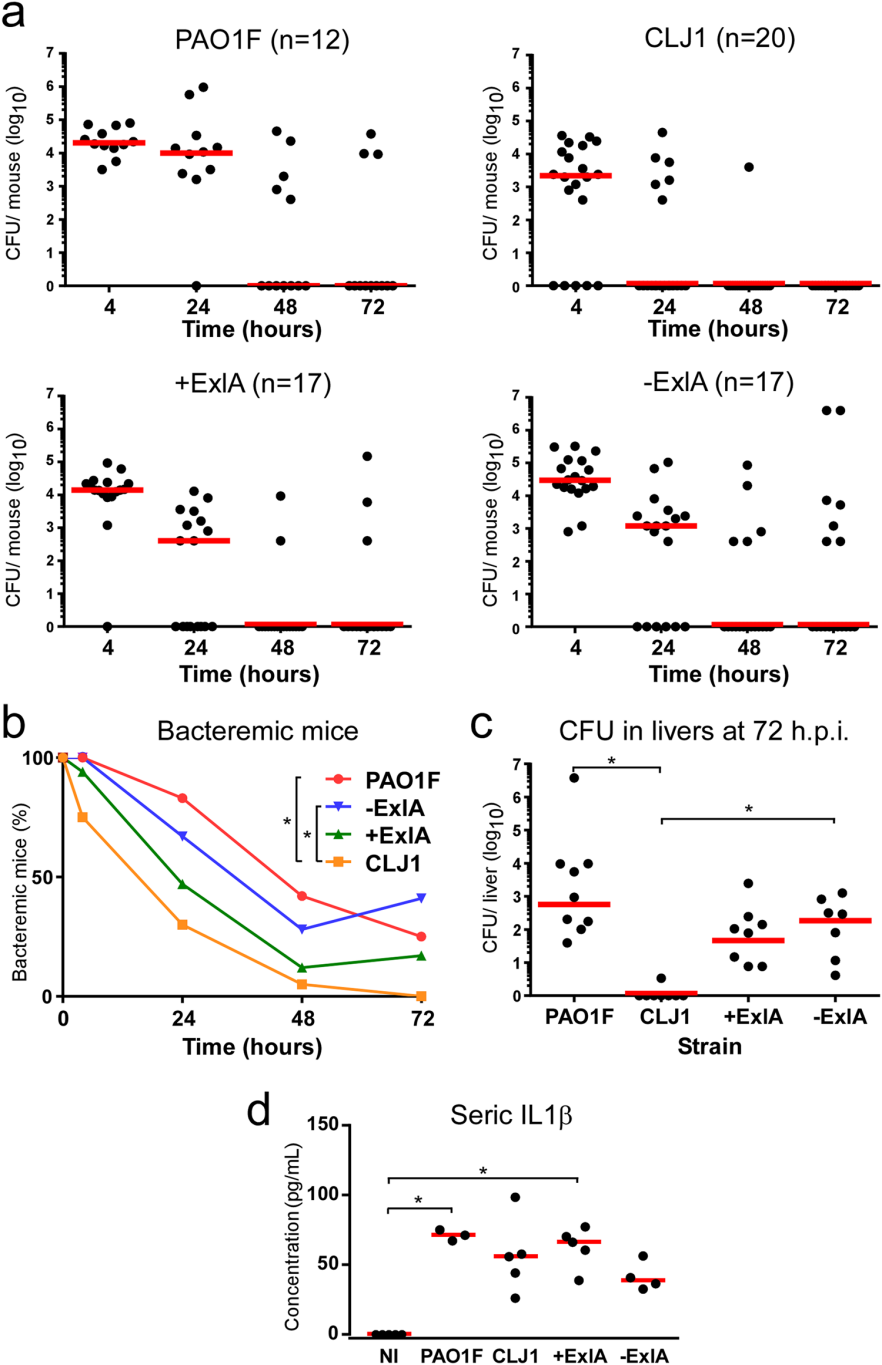
Bacterial survival in blood and liver colonisation in a bacteremic model. Bacteria (1 × 10^7^) from CLJ1, PAO1F, PAO1FΔT3SS::exlBA (+ExlA), PAO1FΔT3SS::vector (−ExlA) strains were injected in mouse caudal veins (mouse numbers are shown after strain names). (**a**) CFU were counted at different time points (4, 24, 48 and 72 h.p.i.) in blood samples and data for each mouse are represented by solid circles for the entire blood compartment. Medians are shown by red bars. (**b**) Percentages of bacteremic mice during time. Statistics: Friedman’s test, p = 0.003; Holm-Sidak’s post-hoc test, *p < 0.05. (**c**) CFU calculated for the entire livers at 72 h.p.i. Statistics: Kruskal-Wallis’s test, p < 0.001; Dunn’s post-hoc test, *p < 0.05. (**d**) Seric levels of IL-1β. Statistics: Kruskal-Wallis’s test, p < 0.004; Dunn’s post-hoc test, *p < 0.05.

We went on to examine the capacity of these bacteria to survive in the blood. Sub-lethal amounts of bacteria (1 10^7^) were injected into the mouse caudal veins and CFU were counted on blood samples at different time points (Fig. 4a). There was a rapid decrease in bacterial burdens of all mice that was more pronounced for CLJ1. The percentages of bacteremic animals were calculated from these data (Fig. 4b), and were significantly different between PAO1F- and CLJ1-infected mice, as well as between −ExlA- and CLJ1-infected mice. Thus, CLJ1 is rapidly cleared from the blood stream. The production of ExlA did not confer to bacteria a significant advantage of persistence in the blood stream, as opposed to lung tissue. The CFU in spleens and livers were counted at 72 h.p.i. No or very few CFU were detected in the spleen, indicating that this organ is not targeted in blood-borne infections (data not shown). The livers were colonised by bacteria, except for CLJ1, which was barely detected in this organ, in good agreement with our blood measurements (Fig. 4c). The data showed significant differences in liver bacterial burdens between CLJ1 and either PAO1F or −ExlA strains. Taken together, the results show that survival in the blood and colonisation of the liver are mainly depend upon the bacterial genetic background and neither ExlA secretion nor the presence of T3SS. The cytokine response was evaluated in this model by IL-1β dosage at 48 h.p.i. in the sera (Fig. 4d). All strains triggered IL-1β release in the blood, although this effect was only statistically significant for PAO1F and +ExlA. IL-1β concentrations were not dramatically different between the strains, as opposed to the results in the BALs (Fig. 3f).

## Discussion

In this paper, we provide the first description of tissue damage induced by ExlA toxin. The general deterioration of CLJ1- or +ExlA-infected lungs and the rupture of the epithelial and endothelial barriers explain why these bacteria can disseminate in the mouse body without a T3SS, although this apparatus was previously shown to be required for bacterial dissemination^6, 9, 10, 16, 17^. We also confirmed that bacteria lacking a functional T3SS do not proliferate in the lung. Importantly, our study revealed that ExlA secretion compensated for the lack of T3SS for dissemination of bacteria in the mouse body and their proliferation in lungs. The destruction of the alveolar tissue probably allows passive diffusion of CLJ1 into the blood, as this bacterium neither swims, swarms nor twitches in laboratory conditions^13^. Although no dramatic histological damage was noted by TEM in PAO1F-infected lungs, the significant increase of proteins in BALs and the spreading of bacteria in the mouse body confirmed that the alveolo-capillary barrier was functionally impaired as previously reported by others for similar *P. aeruginosa* subtypes^9, 18, 19^.

As revealed by the bacterial division rates, the lung offers adequate conditions for rapid CLJ1 proliferation. This is likely a consequence of the lesser neutrophil recruitment in lungs infected by CLJ1^11^. In pulmonary infections with classical *P. aeruginosa* strains, neutrophils are massively targeted to lung alveoli as a result of abundant pro-inflammatory cytokine secretion, which is particularly true for BALB/c mice that have the capacity to mount an efficient immune response following *P. aeruginosa* infection^15, 20, 21^. As revealed here, CLJ1 did not induce a substantial release of five major pro-inflammatory cytokines (IL-1β, TNF-α, IL-12p70, IL-17 and IFN-γ), as opposed to PAO1F. Likewise, the lack of appropriate inflammatory response following CLJ1 infection, likely attributable to deficient cytokine secretion, leaves the way open to bacterial proliferation, virulence and spreading to other organs. In general, bacterial pathogens isolated from chronic infections are relatively silent towards the innate immune system; in this respect, CLJ1 is not an exception to the rule.

Disseminating bacteria were rarely detected in the blood, suggesting that they rapidly colonised and accumulated in target organs, notably in liver and spleen. When high bacterial loads were directly injected into the blood, bacterial counts rapidly decreased in this compartment, further showing that blood is not a favourable environment for *P. aeruginosa*, whatever the genetic background and the virulence factors are. Previously, Vance *et al*.^10^ showed a T3SS− dependence of bacterial survival in blood; however these authors used neutropenic mice, which compromises the phagocytic activity in the blood compartment and eventually bacterial clearance.

Importantly, in the bacteremic model, CLJ1 was more rapidly eliminated than PAO1F and was not detected in the liver, which is in sharp contrast with its disseminating behaviour when introduced in the lung. This feature suggests that CLJ1 is less equipped to resist to phagocytosis when immune cells are in proximity and poses the question of what are the variations in immune cell activation when bacteria originate from primary solid organs or from blood-borne infections, and how bacteria are transported in the blood thereafter. Circulating neutrophils as well as macrophages in the liver (known as Kupffer cells), constituting the first immune rampart in bacteremia, efficiently eliminate circulating bacteria^22^. Liver is known to be the first organ in which bacteria accumulate and are captured by macrophages or recruited neutrophils; bacteria are thus detected mostly in this organ whatever the origin of infection is. Recently, Broadley *et al*.^23^ reported that platelet-bound bacteria are not killed by macrophages or neutrophils, thereby escaping fast clearance in the liver or blood, but accumulate in the spleen, activating adaptive immunity and eventually dying at lower rate (the “slow clearance” track). Hence, it is possible that CLJ1 would be phagocytosed rapidly in the blood or in the liver (fast-clearance track) in the bacteremic model, because it is not associated with platelets, as opposed to strains with PAO1F background. In contrast, when disseminating from the lung, CLJ1 may enter both tracks because of partial association with platelets.

Interestingly, others previously reported that intravenous injection in mice of *P. aeruginosa* strain PA103 (injecting exotoxins U and T via its T3SS) did not induce a septic shock as opposed to lung instillation, although the concentration of circulating bacteria was greater in the bacteremia model^24^. Notably, the circulatory levels of some inflammatory mediators were different between the two models. Thus, the host response to *P. aeruginosa* was diverse whether bacteria were injected intravenously or in the lung; our results confirm this notion.

Surprisingly, following lung infection, −ExlA bacteria were found in the brain in similar numbers as the three other strains. Thus, some −ExlA bacteria may have circulated in the bloodstream and could home in the brain without being killed by phagocytes. Alternatively, bacteria may have colonised the brain from the nasal cavity using the olfactory and trigeminal nerves, as it has been described for other bacterial species^25^, thereby escaping the immune system. To our knowledge, this hypothesis has never been investigated for *P. aeruginosa*.

Some previously described pore-forming toxins were similarly shown to compromise the epithelial and endothelial layers leading to spreading of bacteria into the body and diffusion of blood components into the primary infection site (for a review, see ref. 26). Several death pathways induced by pore-forming toxins have been reported^26^, however, the mechanisms leading to host-plasma membrane rupture induced by ExlA, or the closely related ShlA toxin, are currently unknown. Membrane rupture is quite synchronous for infected cells of the same culture and is rapidly exhaustive. Importantly, similar figures of necrotic death were observed for pneumocytes and endothelial cells in the lungs infected by ExlA-positive bacteria. Thus, the mucus at the epithelial surface, the mucociliary escalator of the bronchi, the presence of immune cells and the highly organised structure of the lung alveoli could not prevent massive necrotic death of the main pulmonary cell types, i.e. pneumocytes and endothelial cells. This is in line with the symptoms of the patient from whom CLJ1 was derived, who suffered from haemorrhagic pneumonia, likely reflecting pulmonary lesions^11^. Moreover, numerous unruptured erythrocytes could be observed by TEM within the mouse alveoli, further suggesting that ExlA does not target this cell type, as previously shown for human erythrocytes^13^. The state of neutrophils was more difficult to appreciate by TEM, because of the polymorphic and moving nature of these cells, but the identified neutrophils did not exhibit signs of necrosis when ExlA-positive bacteria were present.

In conclusion, the ExlA-expressing strain CLJ1 displayed a devastating activity in infected lungs, stronger than the reference PAO1F strain. Here we provide compelling evidence showing that ExlA confers to the bacteria the capacity to translocate in the bloodstream and to produce metastatic infections of essential organs. We link this virulence characteristic of ExlA to its property to trigger cytotoxic effects without alarming the innate immune system. The characterization of the host signalling pathways induced by ExlA, and leading to epithelial/endothelial cell necrosis, is underway.

## Materials and Methods

### Ethical statement

All protocols in this study were conducted in strict accordance with the French guidelines for the care and use of laboratory animals. The protocols for mouse infection were approved by the animal research committee of the institute (CETEA, project number 13-024, CIEPAL project number PEA311) and the French Ministry of Research.

### *P. aeruginosa* strains and culture

The strains used in this study are described in Table 1. Bacteria were grown in liquid LB medium at 37 °C with agitation until the cultures reached an optical density value of 1.0. No arabinose was included in the culture of +ExlA bacteria to maintain the secreted −ExlA amounts equivalent in +ExlA and CLJ1 strains^11^.

**Table 1:** *P. aeruginosa* strains used in this work.

Strains	Description	Abbreviations	Refs
PAO1F (RP1831)	Wild-type laboratory strain T3SS+ (*exoS,T,Y*)	PAO1F	27
CLJ1	Clinical strain T3SS−, ExlA+	CLJ1	11
PAO1FΔ*T3SS*::*exlBA*	PAO1F T3SS− (Δ*pscD*) ExlBA+	+ExlA	11
	The strain is devoid of T3SS and possesses one copy of *exlBA* locus in the chromosome		
PAO1FΔ*T3SS*::empty vector	control: PAO1F T3SS− (Δ*pscD*)	−ExlA	11
	The strain is devoid of T3SS		

### Mouse pulmonary infection

Pathogen-free BALB/c female mice (8–10 weeks) were obtained from Harlan Laboratories and housed in the institute animal care facility. Bacteria from exponential growth (OD = 1.0) were centrifuged and resuspended in sterile PBS at 0.85 × 10^8^ per mL. Mice were anesthetized by intraperitoneal administration of a mixture of xylazine (10 mg.Kg^−1^) and ketamine (50 mg.Kg^−1^). Then, 30 μL of bacterial suspension (2.5 × 10^6^) were deposited into the mouse nostrils. Mice were euthanized by CO_2_ inhalation at indicated times.

To study bacterial dissemination, various organs were isolated on euthanized mice. Blood was sampled from jugular vein. Spleen, liver, kidney, brain and lungs were homogenized in PBS with a Polytron. *P. aeruginosa* CFU were determined by plating of serial dilutions and colony counting on Pseudomonas Isolation Agar (Difco) plates. The CFU were calculated for the total organs.

In independent experiments, bronchoalveolar lavages (BAL) were performed by injection of 0.5 mL PBS (3 times) on euthanized and intubated mice at 18 h.p.i. Collected fluids were pooled, centrifuged and the supernatants and the pellets were aliquoted, snap-frozen and stored at −80 °C. Measurement of IL1-β, TNF-α and IFN-γ concentrations on BAL supernatants was performed using the LEGENDplex system (BioLegend) on a FACSCalibur cytometer and data were analysed with the LEGENDplex Data Analysis Software. Seric levels of IL-1β were measured with the IL-1β ELISA kit from R&D. Lactate dehydrogenase (LDH) activity in BAL supernatants was assayed using the Cytotoxicity Detection kit from Roche. The presence of actin was determined by Western blot analysis on 6 µL of BAL supernatants, using an anti-β-actin antibody from Sigma-Aldrich. Protein concentration of BAL supernatants was measured using the BCA kit from Thermo Scientific. The BAL pellets were resuspended in 200 µL of lysis buffer and their MPO activity was determined using the Myeloperoxidase fluorimetric kit from Enzo.

### Mouse bacteremia

Female BALB/c mice (6–8 weeks old) were purchased from Janvier (Le Genest St Isle, France). Bacteria from exponential growth (OD = 1.0) were centrifuged, washed once with sterile PBS and resuspended in sterile PBS to an optical density value of 0.3. Mice were injected i.v. with 50 µL of this suspension (1 × 10^7^ CFU of bacteria per mouse).

For the determination of bacteremia, blood (5 µL) was collected from the tail vein at indicated times post-infection, diluted in sterile PBS, plated on Pseudomonas Isolation Agar. CFU were calculated as the number of bacteria for each mouse. At 72 h.p.i., mice were euthanized, liver and kidney were homogenized in PBS using a Precellys® Evolution homogenizer (Bertin Corp, Rockville, MD), and CFU were determined by plating as above.

### Histological analysis

Similar experiments were conducted to examine the histology of infected organs. Organs were fixed, paraffin-embedded and sections were prepared using hematoxylin-eosin staining for morphological examination, or for bacterial labelling with anti-LPS antibody (Acris Antibodies), with Yo-Pro (Molecular Probes) counterstaining of nuclei. Slides were observed under an Axioplan Zeiss microscope.

### Transmission electron microscopy

For electron microscopy, euthanized mice at 18 h.p.i. were rapidly perfused intracardiacally with NaCl 0.9%/heparin 20 U/mL and then with a fixative solution (paraformaldehyde 4%/glutaraldehyde 2%). Lungs were minced in small pieces (about 1 mm3) and further fixed in fixative solution (paraformaldehyde 2%/glutaraldehyde 0.2% in 0.1 M cacodylate buffer) for 24 h at 4 °C. Post-fixation was performed during 1 hour in osmium solution (1% osmium, 1.5% potassium hexaferrocyanate in 0.1 M PHEM buffer) under shaking. After water washes, samples were stained with uranyl acetate (5% in water) under shaking. Dehydration occurred in graded series of ethanol (50 to 100%) before substitution and impregnation in Embed 812 resin (EPON substitute, EMS). After 48 h of polymerisation at 65 °C, the blocs were ready to be trimmed and cut. Ultramicrotome UC7 (Leica) was used to generate 1-µm sections, collected on glass slides, for localisation of regions of interest by optical imaging (after toluidine staining). For TEM imaging, 80-nm sections, collected on formvar–coated copper grids, were observed using a FEI Tecnai G2 Spirit BioTwin transmission electron microscope operating at 120 kV using an Orius SC1000B CCD camera. Two mice were used per bacterial strain.

### Statistics

Statistical tests were performed using SigmaPlot software (Systat Software Inc.). Normality (Shapiro-Wilk’s test) and equal variance tests were applied on data. Multicomparison tests were used thereafter: ANOVA, when the data passed the normality and variance tests, and Kruskal-Wallis or Friedman on ranks, when they did not. They were followed by pairwise comparison tests (Holm-Sidak’s or Dunn’s), as indicated in figure legends.

## Electronic supplementary material

Supplementary Information

## References

[1] Gellatly SL, Hancock RE (2013). Pseudomonas aeruginosa: new insights into pathogenesis and host defenses. Pathogens and disease.

[2] Williams BJ, Dehnbostel J, Blackwell TS (2010). Pseudomonas aeruginosa: host defence in lung diseases. Respirology.

[3] Deng Q, Barbieri JT (2008). Molecular mechanisms of the cytotoxicity of ADP-ribosylating toxins. Annual review of microbiology.

[4] Hauser AR (2009). The type III secretion system of Pseudomonas aeruginosa: infection by injection. Nature reviews.

[5] Sawa T (2014). The molecular mechanism of acute lung injury caused by Pseudomonas aeruginosa: from bacterial pathogenesis to host response. J Intensive Care.

[6] Allewelt M, Coleman FT, Grout M, Priebe GP, Pier GB (2000). Acquisition of expression of the Pseudomonas aeruginosa ExoU cytotoxin leads to increased bacterial virulence in a murine model of acute pneumonia and systemic spread. Infection and immunity.

[7] Hauser AR (2002). Type III protein secretion is associated with poor clinical outcomes in patients with ventilator-associated pneumonia caused by Pseudomonas aeruginosa. Critical care medicine.

[8] Le Berre R (2011). Relative contribution of three main virulence factors in Pseudomonas aeruginosa pneumonia. Critical care medicine.

[9] Shaver CM, Hauser AR (2004). Relative contributions of Pseudomonas aeruginosa ExoU, ExoS, and ExoT to virulence in the lung. Infection and immunity.

[10] Vance RE, Rietsch A, Mekalanos JJ (2005). Role of the type III secreted exoenzymes S, T, and Y in systemic spread of Pseudomonas aeruginosa PAO1 *in vivo*. Infection and immunity.

[11] Elsen S (2014). A type III secretion negative clinical strain of Pseudomonas aeruginosa employs a two-partner secreted exolysin to induce hemorrhagic pneumonia. Cell Host Microbe.

[12] Huber P, Basso P, Reboud E, Attree I (2016). Pseudomonas aeruginosa renews its virulence factors. Environ Microbiol Rep.

[13] Reboud E (2016). Phenotype and toxicity of the recently discovered exlA-positive Pseudomonas aeruginosa strains collected worldwide. Environ Microbiol.

[14] Ader F (2007). Inhaled nitric oxide increases endothelial permeability in Pseudomonas aeruginosa pneumonia. Intensive care medicine.

[15] De Simone M (2014). Host genetic background influences the response to the opportunistic Pseudomonas aeruginosa infection altering cell-mediated immunity and bacterial replication. PloS one.

[16] Kudoh I, Wiener-Kronish JP, Hashimoto S, Pittet JF, Frank D (1994). Exoproduct secretions of Pseudomonas aeruginosa strains influence severity of alveolar epithelial injury. The American journal of physiology.

[17] Rangel SM, Diaz MH, Knoten CA, Zhang A, Hauser AR (2015). The Role of ExoS in Dissemination of Pseudomonas aeruginosa during Pneumonia. PLoS pathogens.

[18] Ader F (2005). Alveolar response to Pseudomonas aeruginosa: role of the type III secretion system. Infection and immunity.

[19] Lee VT, Smith RS, Tummler B, Lory S (2005). Activities of Pseudomonas aeruginosa effectors secreted by the Type III secretion system *in vitro* and during infection. Infection and immunity.

[20] Morissette C, Skamene E, Gervais F (1995). Endobronchial inflammation following Pseudomonas aeruginosa infection in resistant and susceptible strains of mice. Infection and immunity.

[21] Tam M, Snipes GJ, Stevenson MM (1999). Characterization of chronic bronchopulmonary Pseudomonas aeruginosa infection in resistant and susceptible inbred mouse strains. American journal of respiratory cell and molecular biology.

[22] Jenne CN, Kubes P (2013). Immune surveillance by the liver. Nat Immunol.

[23] Broadley SP (2016). Dual-Track Clearance of Circulating Bacteria Balances Rapid Restoration of Blood Sterility with Induction of Adaptive Immunity. Cell Host Microbe.

[24] Kurahashi K (1999). Pathogenesis of septic shock in Pseudomonas aeruginosa pneumonia. The Journal of clinical investigation.

[25] Dando SJ (2014). Pathogens penetrating the central nervous system: infection pathways and the cellular and molecular mechanisms of invasion. Clin Microbiol Rev.

[26] Los FC, Randis TM, Aroian RV, Ratner AJ (2013). Role of pore-forming toxins in bacterial infectious diseases. Microbiol Mol Biol Rev.

[27] Bleves S, Soscia C, Nogueira-Orlandi P, Lazdunski A, Filloux A (2005). Quorum sensing negatively controls type III secretion regulon expression in Pseudomonas aeruginosa PAO1. Journal of bacteriology.

