# Supplementary material for: Pseudomonas aeruginosa Exolysin promotes bacterial growth in lungs, alveolar damage and bacterial dissemination
Source: Sci Rep. 2017 May 18;7:2120. doi: 10.1038/s41598-017-02349-0 (PMC5437091; doi:10.1038/s41598-017-02349-0)
Supplement: Supplementary file 1 — Supplementary Information [file 41598_2017_2349_MOESM1_ESM.pdf]

Pseudomonas aeruginosa Exolysin promotes bacterial growth in lungs, alveolar damage and bacterial dissemination

Stéphanie Bouillot, Patrick Munro, Benoit Gallet, Emeline Reboud, François Cretin, Guillaume Golovkine, Guy Schoehn, Ina Attrée, Emmanuel Lemichez, and Philippe Huber

Supplementary Information

SUPPLEMENTARY METHODS

Confocal microscopy. Cells were imaged using a confocal spinning-disk inverted microscope (Nikon TI-E Eclipse) equipped with an Evolve EMCCD camera. Images were acquired using an illumination system from Roper Scientific (iLasPulsed) with a CFI Plan APO VC oil-immersion objective (60X, N.A 1.4). Z-series were generated using a motorized Z-piezo stage (ASI) by acquiring images with a step size of 0.4 µm.

Western blot analysis. IL-1β and β-actin were detected using antibodies from SantaCruz and Sigma, respectively. Luminescent signals were revealed using a ChemiDoc (BioRad) and only non-saturated signals are shown.

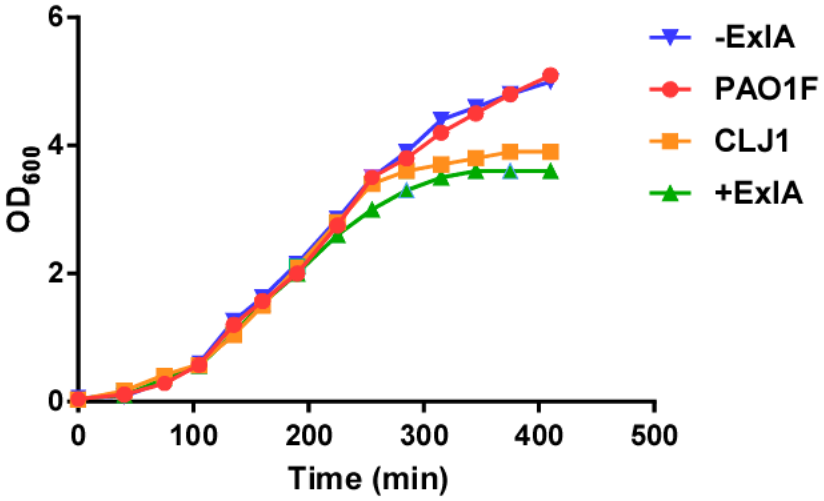

Supplementary Fig. 1: Bacterial growth curves in LB medium

Bacteria from overnight cultures were seeded at OD_{600} = 0.01 and the optical densities were measured until 400 min.

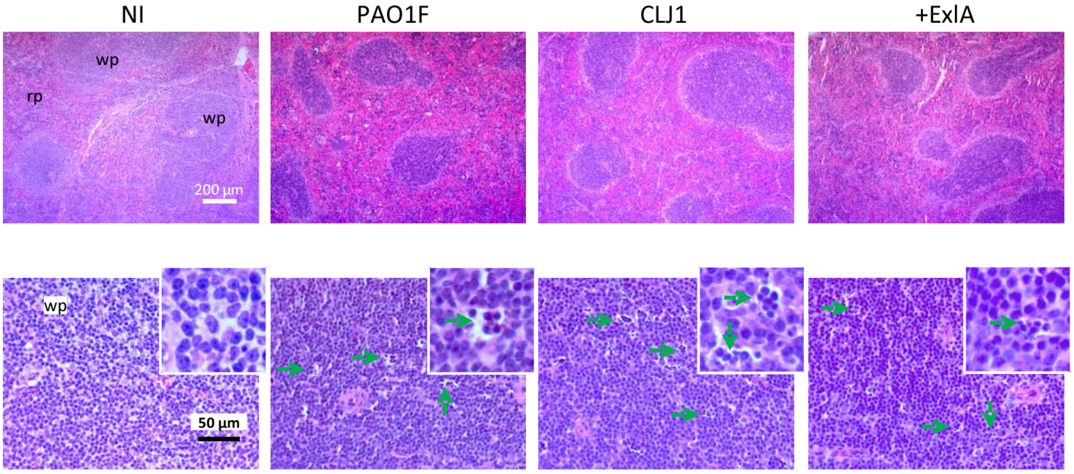

Supplementary Fig. 2: Histology of spleens after pulmonary infections with PAO1F, CLJ1 and +ExlA strains or from not infected mice (NI). Pulmonary infection procedure is described in Fig. 1 legend. Spleens were harvested at 23 h.p.i., and processed for paraffin inclusion. Sections were stained with hematoxylin-eosin and observed with an Axioplan microscope (Zeiss). Pictures were taken with a color camera (Spot-RT from Diagnostic instruments). At low magnification (above), the images show the red pulp (rp) hyperhaemia after infection with all three strains, as indicated by the darker red staining. At high magnification (below), a white pulp (wp) hyperplaesia can be observed in infected spleens (increased nuclei density), together with groups of aggregated apoptotic B cells (arrows and magnified inserts). These figures are characteristic of immunoreactive spleens in inflammatory conditions.

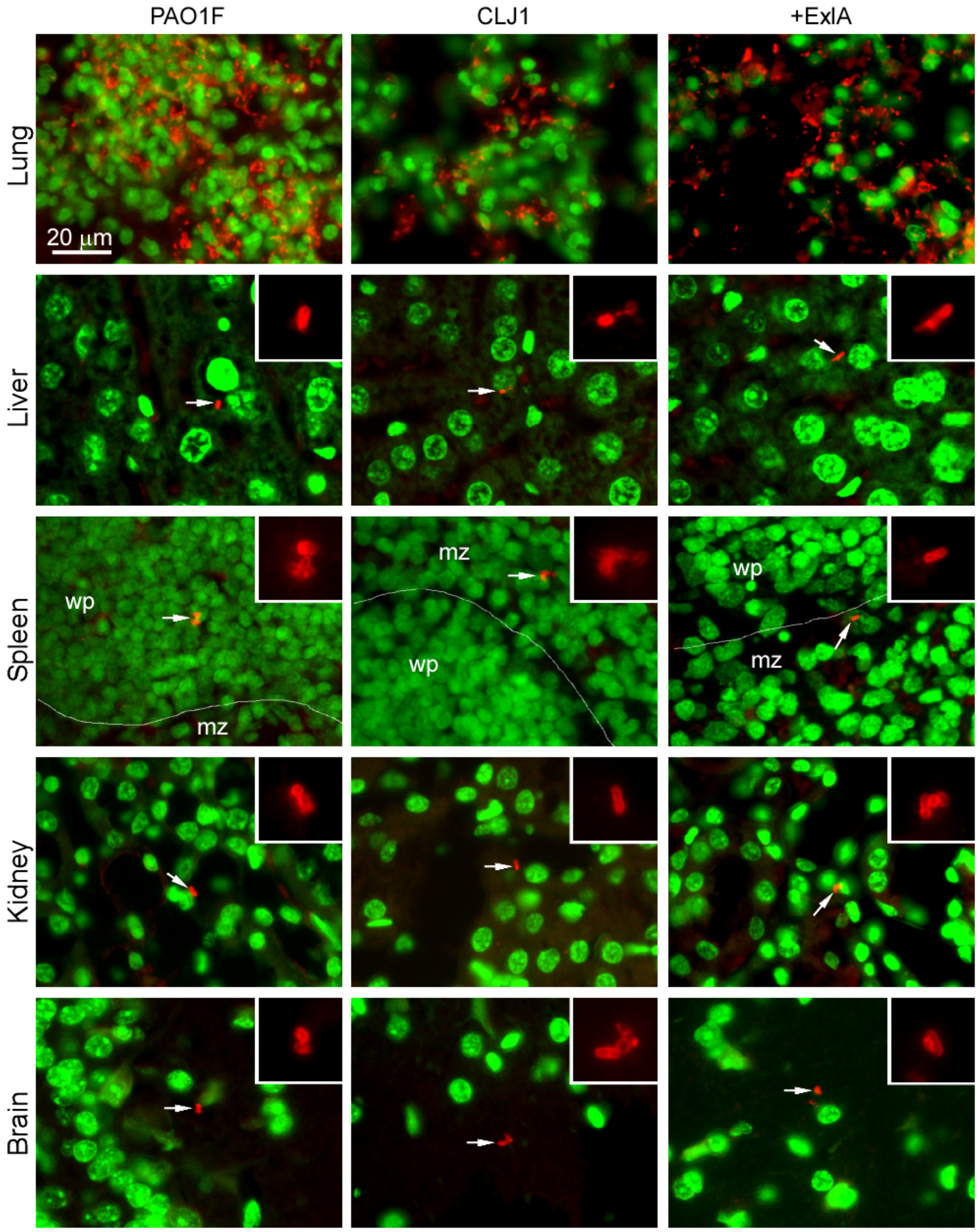

Supplementary Fig. 3: Localisation of bacteria in infected organs. Mice were subjected to lung infection with different bacterial strains (2.5 10^6) as indicated. After 18 h.p.i., mice were euthanised and organs (indicated on the left) were paraffin-embedded. Sections were labelled with anti-LPS antibody for bacteria staining (red) and with Yo-Pro (green) for nuclei straining. Bacteria (arrows) were detected in lung alveoli, hepatic lobules, white pulp (wp) and marginal zone (mz) of spleen, intertubular space of kidney and brain parenchyma.

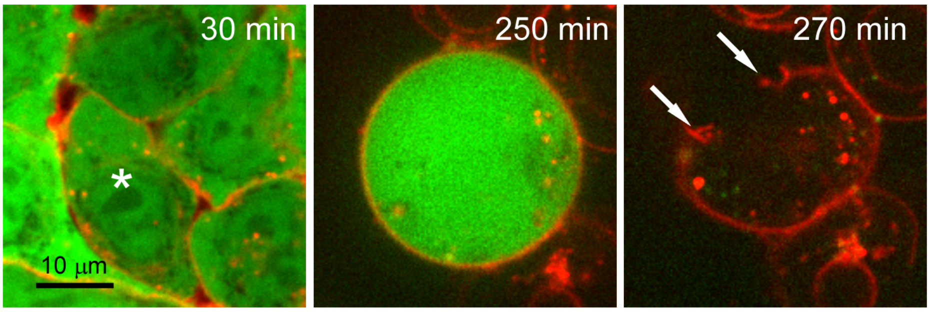

Supplementary Fig. 4: Cell membrane rupture induced by CLJ1
Confluent A549-EGFP cells (green) were incubated with WGA-alexa 647 (red) to label the plasma membranes. Cells were infected with CLJ1 at MOI of 10 and were observed by confocal microscopy in real time. Images were acquired every 10 min. The figure shows representative images of one z-plane at indicated time points post-infection. (*) indicates the cell shown in the next panels. Arrows show the edges of the ruptured plasma membrane.

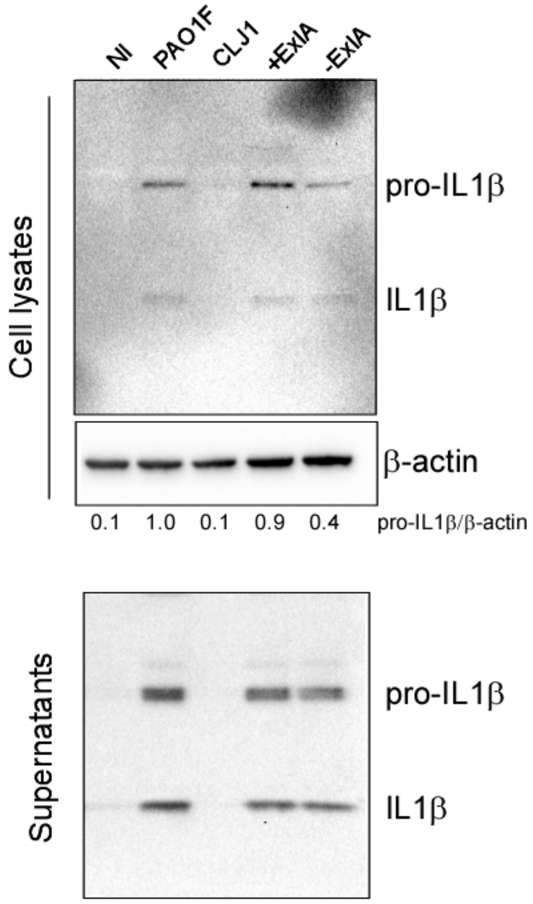

Supplementary Fig. 5: ILβ expression and secretion in infected A549 cells

A549 cells were either uninfected (NI) or incubated with PAO1F, CLJ1, +ExlA or -ExlA at MOI 10. Cell supernatants were harvested at 3 h.p.i. and cell lysates were prepared. All samples were analysed by Western blot to reveal IL1β, and β-actin for the cell lysates. Pro-ILβ/ β-actin signal ratios are shown below the lanes of lysate samples. Pro-IL1b signals were detected in the supernatants, indicating that some cells have lysed.
